# Gastric leiomyosarcoma and diagnostic pitfalls: a case report

**DOI:** 10.1186/s12893-018-0393-4

**Published:** 2018-08-17

**Authors:** Anis Hasnaoui, Raja Jouini, Dhafer Haddad, Haithem Zaafouri, Ahmed Bouhafa, Anis Ben Maamer, Ehsen Ben Brahim

**Affiliations:** 1 Department of General Surgery, Habib Thameur Hospital, Tunis El Manar University, Ali Ben Ayed Street 2037 Montfleury, Tunis, Tunisia; 2 Department of Histopathology and Cytology, Habib Thameur Hospital, Tunis El Manar University, Tunis, Tunisia

**Keywords:** Leiomyosarcoma, Gastric, Bleeding, H-caldesmon, KIT, DOG1, GIST

## Abstract

**Background:**

Since the advent of immunohistochemistry for the diagnosis of stromal tumours, the incidence of leiomyosarcomas has significantly decreased. Nowadays, gastric leiomyosarcoma is an exceptionally rare tumour. We report the second case in the English literature of gastric leiomyosarcoma revealed with massive bleeding and hemodynamic instability and diagnostic pitfalls that we encountered.

**Case presentation:**

A 63-year-old woman, with 2 years’ history of dizziness and weakness probably related to an anaemic syndrome, presented to the emergency room with hematemesis, melena and hemodynamic instability.

On examination, she had conjunctival pallor with reduced general condition, blood pressure of 90/45 mmHg and a pulse between 110 and 120 beats per minute. On digital rectal examination, she had melena. Laboratory blood tests revealed a haemoglobin level at 38 g/L.

The patient was admitted to the intensive care department. After initial resuscitation, transfusion and intravenous Omeprazole continuous infusion, her condition was stabilized. She underwent upper gastrointestinal endoscopy showing a tumour of the cardia, protruding in the lumen with mucosal ulceration and clots in the stomach. Biopsies were taken. Histological examination showed interlacing bundles of spindle cells, ill-defined cell borders, elongated hyperchromatic nuclei with marked pleomorphism and paranuclear vacuolization. Immunohistochemistry showed positivity for Vimentine, a strong and diffuse immunoreactivity for smooth muscle actin (SMA). Immunoreactivities for KIT and DOG1 were doubtful.

Computed tomography scan revealed a seven-cm tumour of the cardia, without adenopathy or liver metastasis.

The patient underwent laparotomy. A total gastrectomy was performed without lymphadenectomy. Post-operative course was uneventful.

Histological examination of the tumour specimen found the same features as preoperative biopsies with negative margins. We solicited a second opinion of an expert in a reference centre for sarcomas in France, who confirmed the diagnosis of a high grade gastric leiomyosarcoma.

**Conclusion:**

Gastric leiomyosarcoma is a rare tumour. Diagnosis is based on histological examination with immunohistochemistry, which could be sometimes confusing like in our case. The validation of a pathological expert is recommended.

## Background

Gastrointestinal stromal tumours (GISTs) were considered to be of smooth muscle origin. They were misdiagnosed as leiomyomas and leiomyosarcomas. Since the advent of immunohistochemistry for the diagnosis of stromal tumours, the incidence of leiomyosarcomas has significantly decreased. Nowadays, gastric leiomyosarcoma is an exceptionally rare tumour [1]. Discovery of this tumour is generally made at a late stage as its growth is often insidious. Diagnosis relies on accurate histological examination with immunohistochemistry, as treatment and prognosis differ widely between different types of mesenchymal tumours.

We present the case of a gastric leiomyosarcoma revealed by a massive upper gastrointestinal bleeding and diagnostic pitfalls that we encountered.

## Case presentation

A 63-year-old woman, with 2 years’ history of dizziness and weakness probably related to an anaemic syndrome, presented to the emergency room with hematemesis, melena and hemodynamic instability. There was no history of chronic liver disease, dyspepsia, ulcer disease, nonsteroidal anti-inflammatory drugs or aspirin use.

On examination, she had conjunctival pallor with reduced general condition, blood pressure of 90/45 mmHg and a pulse between 110 and 120 beats per minute. On digital rectal examination, she had melena. There were no abdominal wall varices, no hepatomegaly, and no palpable mass or adenopathy.

Laboratory blood tests revealed a haemoglobin level at 38 g/l with haematocrit at 13.4%. The mean corpuscular volume was in the normal range.

**Fig. 1:**
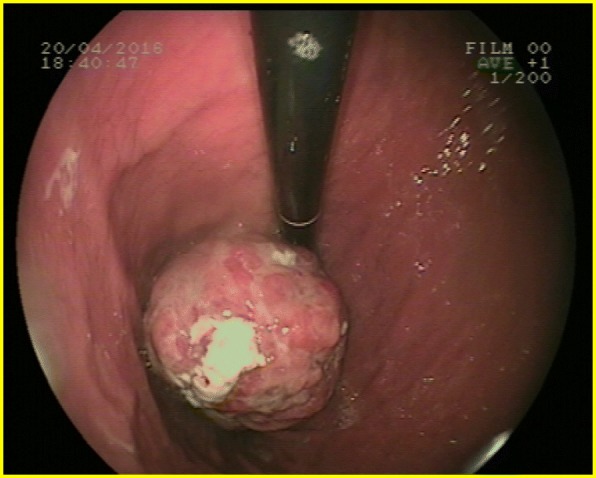
Tumour of the cardia protruding in the gastric lumen

The patient was admitted to the intensive care department. After initial resuscitation, transfusion and intravenous Omeprazole continuous infusion, her condition was stabilized. She underwent upper gastrointestinal endoscopy showing a tumour of the cardia, protruding in the lumen with mucosal ulceration and clots in the stomach (Fig. 1). Biopsies were taken. Histological examination showed interlacing bundles of spindle cells, ill-defined cell borders, elongated hyperchromatic nuclei with marked pleomorphism and numerous mitoses. Immunohistochemistry showed positivity for Vimentine, a strong and diffuse immunoreactivity for SMA. Immunoreactivities for KIT and DOG1 were doubtful.

**Fig. 2:**
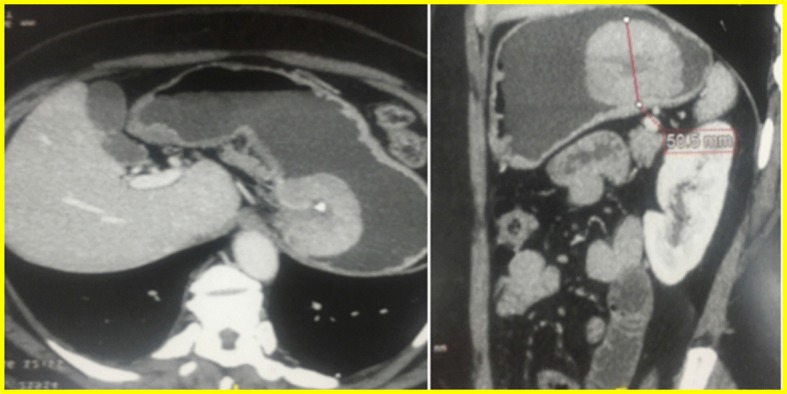
CT scan showing the tumour in the cardia

Computed tomography (CT) scan revealed a seven-cm tumour of the cardia, without adenopathy or liver metastasis (Fig. 2).

After multidisciplinary meeting, we suspected the diagnosis of stromal tumour of the cardia with high risk of re-bleeding and we decided to perform a total gastrectomy.

**Fig. 3:**
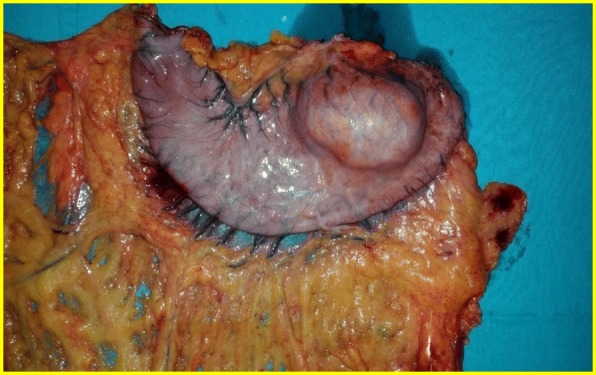
Resection specimen: Total gastrectomy with a nine-cm tumour of the cardia and fundus

The patient underwent laparotomy. There was a nine-cm tumour of the cardia and the fundus, and no sign of peritoneal seeding or liver metastasis. A total gastrectomy was performed without lymphadenectomy (Fig. 3). Post-operative course was uneventful.

**Fig. 4:**
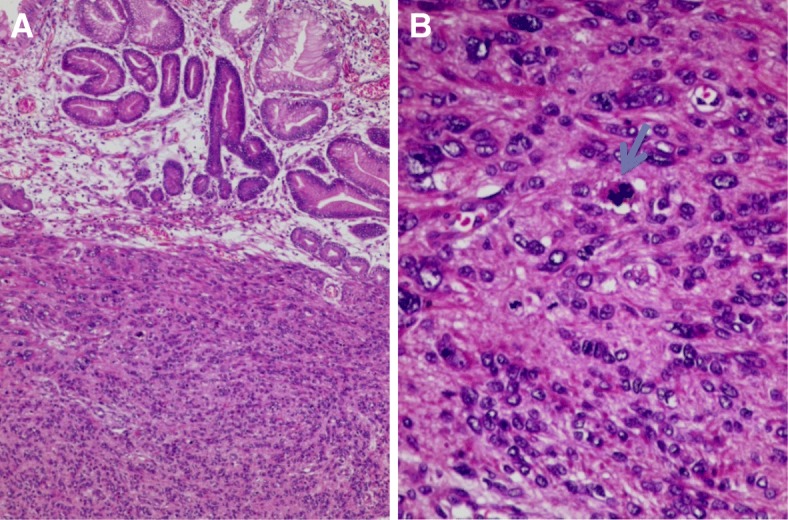
Gastric fusocellular proliferation (**a**) with marked atypia and numerous mitoses (**b**). Arrow shows an abnormal mitotic figure (Haematoxylin and eosin stain)

**Fig. 5:**
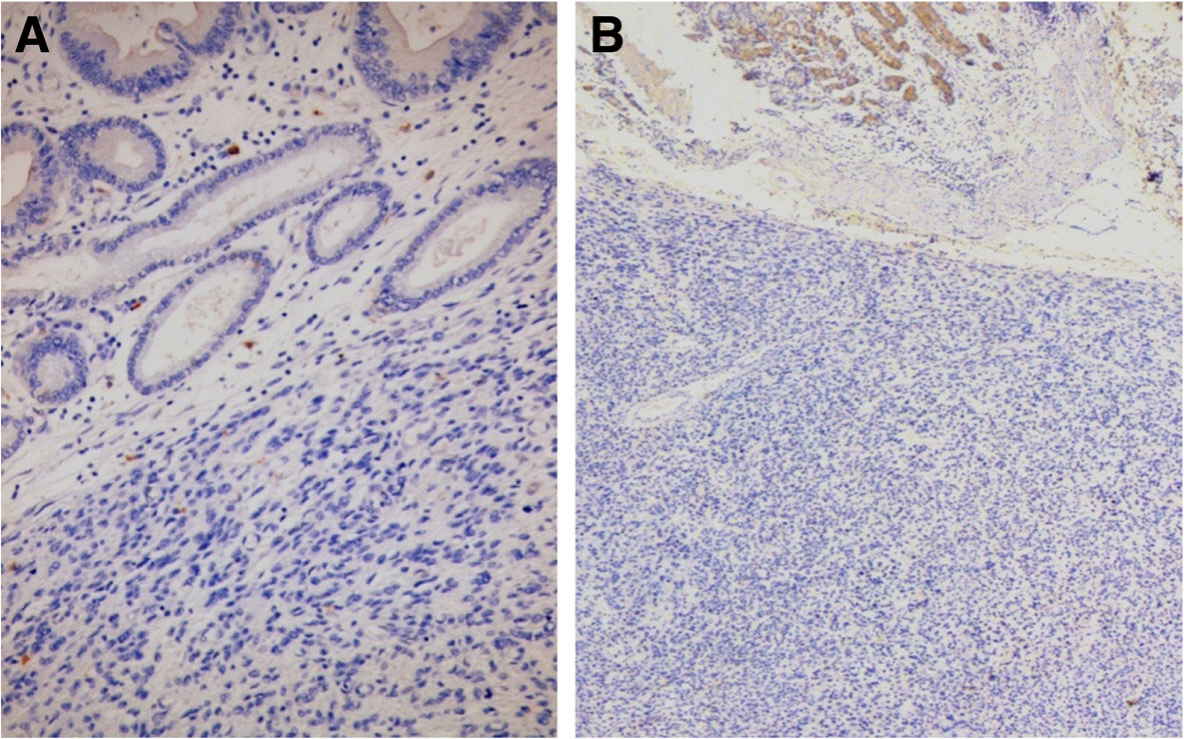
Immunohistochemically, tumor cells are KIT negative, only mast cells are positive (**a**). Tumor cells are also DOG 1 negative (**b**), with normal positivity in gastric epithelium

**Fig. 6:**
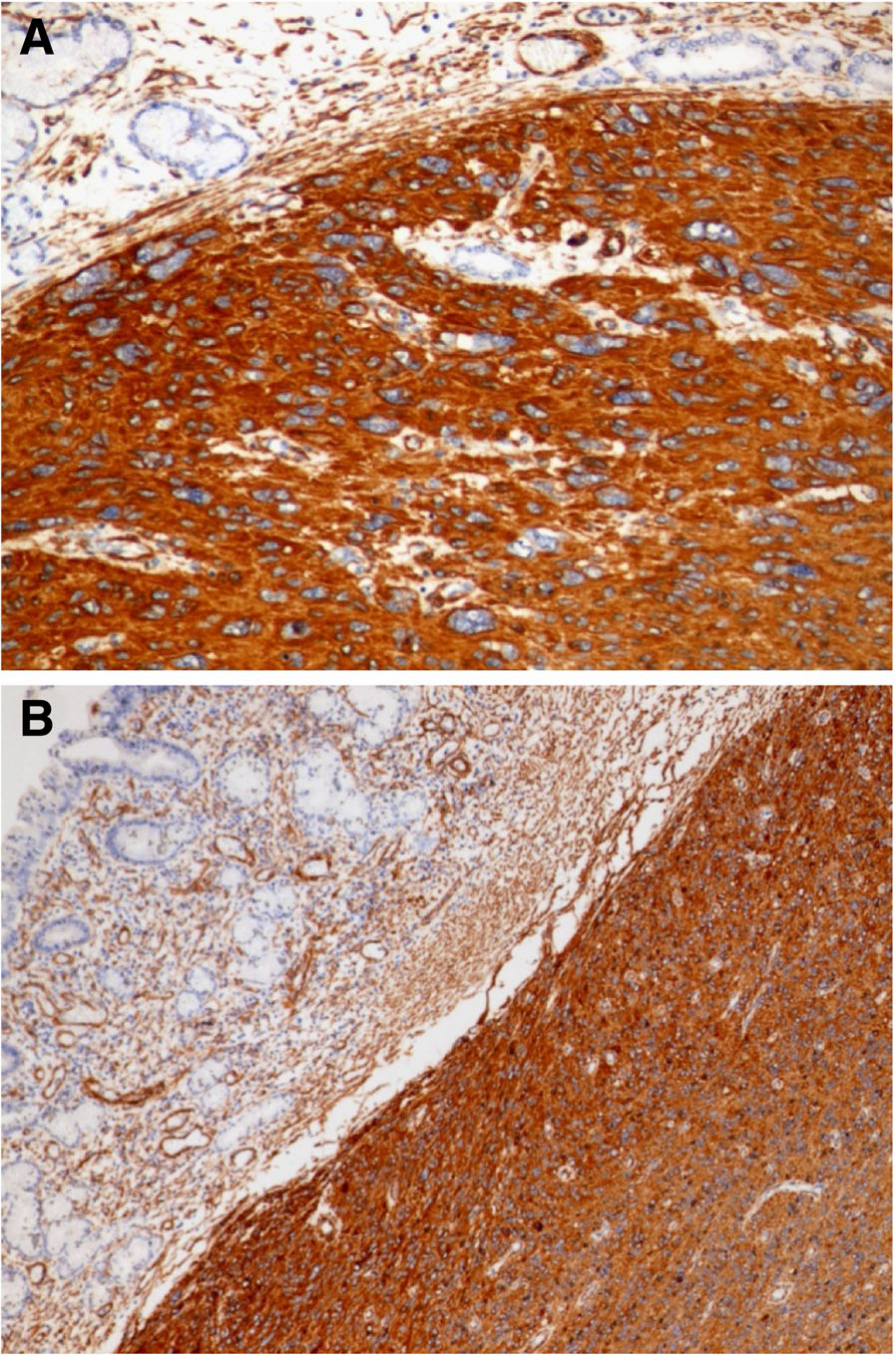
Immunohistochemically, tumor cells express SMA (**a**) and h-caldesmon (**b**)

Histological examination of the tumour specimen found the same features as preoperative biopsies with negative margins (Fig. 4). We solicited a second opinion of an expert in a reference centre for sarcomas in France. Immunohistochemistry showed the following: DOG1 staining was focally positive for some normal cells of Cajal. Otherwise, neoplastic cells were DOG1 -, c Kit - (Fig. 5), CD34 -, smooth muscle actin + and h-caldesmon + (Fig. 6). In conclusion, it was in favour of a high grade gastric leiomyosarcoma.

## Discussion and conclusion

Before the late 90’s, GISTs were misdiagnosed as leiomyomas and leiomyosarcomas [2]. Advances in immunohistochemistry led to the decrease of the incidence of gastric leiomyosarcomas to 1% of all malignant gastric tumours [1, 3, 4]. We report a case of this rare sarcoma with some particularities.

First, gastric leiomyosarcomas are generally insidious since they have a predominant extraluminal component [5]. It may be revealed by gastric outlet obstruction, perforation [6] or bleeding like in our case. Bleeding generally occurs when the tumour erodes the mucosa causing a chronic anaemia. Massive bleeding is not very common. To our knowledge, this is the second case in the literature of gastric leiomyosarcoma with massive bleeding and hemodynamic instability [4].

Second, arising between the muscularis propria and muscularis mucosa layers, diagnosis of leiomyosarcomas relies on histological examination of deep biopsies. Conventional endoscopy usually yields superficial and normal mucosa. Endoscopic ultrasonography, on the other hand, has been proved to be of great sensitivity, up to 97% [1], in the diagnosis of these tumours. It may be required to obtain deep biopsies. In our establishment, this technique was not available. Nevertheless, we succeeded to obtain an adequate sampling with conventional endoscopy. This is may be due to the endoluminal growth of the tumour and mucosal ulceration.

Third, histological examination was not evident. In the first and second pathology reports, based respectively on endoscopic biopsies and resection specimen, we had doubts about the positivity of KIT and DOG1 immunoreactivities. These two markers present the basis for the diagnosis of GISTs. Miettinen et al. declared that sensitivities of DOG1 and KIT were nearly identical: 94.4% and 94.7% [7]. DOG1 is considered the best marker for GISTs with better specificity [8, 9]. More recent studies showed that DOG1 positivity could be detected in neoplastic tissues other than GISTs [10–12]. But, there were no cases of gastric leiomyosarcoma with positive DOG1 staining like ours. In our case, positivity of DOG1 markers resulted in diagnostic confusion, especially that postoperative therapeutic approach will differ depending on whether it is a stromal tumour or not. In such cases, based on Ray-Coquard et al. study [13], the European Society for Medical Oncology (ESMO) recommends the validation of a pathological expert “when the original diagnosis was made outside a reference centre/network” [14]. So, we requested another opinion from an expert in France to confirm the diagnosis.

Finally, the localization of the tumour in the cardia is exceptional and presents a challenge for the surgeon especially if adjacent structures, such as the aorta, are invaded. In fact, surgery is the only curative option for leiomyosarcomas. The type of surgery depends on the size and localization of the tumour [15]. It ranges from a wedge resection to a total gastrectomy with en bloc resection if adjacent organs are invaded. In March 2018, Sato et al. first published a case of a small gastric leiomyosarcoma treated with endoscopic submucosal dissection [16]. Resection margins affect directly the prognosis. Systematic lymphadenectomy is not recommended as leiomyosarcoma have predilection for hematogenous spread and lymph node involvement is rare [6]. In our case, a total gastrectomy was performed rather than partial resection due to the size and localization of the tumour.

In conclusion, gastric leiomyosarcoma is a rare tumour. Diagnosis is based on histological examination with immunohistochemistry, which could be sometimes confusing like in our case. The validation of a pathological expert is recommended. Treatment depends on surgery with a very little place reserved for chemotherapy and radiotherapy in advanced cases [17, 18]. Prognosis is still very poor [1, 4, 19].

## Abbreviations

CT: Computed tomography
ESMO: European Society for Medical Oncology
GIST: Gastrointestinal stromal tumours
SMA: Smooth muscle actin
